# A new silesaurid from Carnian beds of Brazil fills a gap in the radiation of avian line archosaurs

**DOI:** 10.1038/s41598-023-32057-x

**Published:** 2023-04-11

**Authors:** Rodrigo T. Müller, Maurício S. Garcia

**Affiliations:** 1 Centro de Apoio à Pesquisa Paleontológica da Quarta Colônia, Universidade Federal de Santa Maria, Rua Maximiliano Vizzotto, São João do Polêsine, Rio Grande do Sul 598, 97230-000 Brazil; 2 Programa de Pós-Graduação em Biodiversidade Animal, Universidade Federal de Santa Maria, Santa Maria, Rio Grande do Sul 97105-120 Brazil

**Keywords:** Palaeontology, Phylogenetics, Taxonomy, Palaeontology

## Abstract

Comprising the oldest unequivocal dinosauromorphs in the fossil record, silesaurs play an important role in the Triassic radiation of dinosaurs. These reptiles provide the main source of information regarding the ancestral body plan of dinosaurs, as well as the basis for biogeographic models. Nevertheless, the co-occurrence of silesaurs and the oldest unequivocal dinosaurs is rare, which hampers reliable ecological inferences. Here we present the first species of silesaur from the oldest unequivocal dinosaur-bearing beds from Brazil. *Amanasaurus nesbitti* gen. et sp. nov. possesses a unique set of femoral traits among silesaurs, including the oldest occurrence of an anterior trochanter separated by the femoral shaft by a marked cleft. Its femoral length indicates that the new species rivals in size with most coeval dinosaurs. This find challenges the assumption that in faunas where silesaurs and unambiguous dinosaurs co-occurred, silesaurs were relatively smaller. Moreover, the presence of dinosaur-sized silesaurs within ecosystems with lagerpetids, sauropodomorphs and herrerasaurids reinforces the complex scenario regarding the early radiation of Pan-Aves. Silesaurs—independent of their phylogenetic position—persisted during most of the Triassic Period, with its plesiomorphic body size advancing through the dawn of dinosaurs, instead of silesaur lineages decrease in body size through time.

## Introduction

Silesaurs are part of the wide Triassic radiation of archosaurs^1,2^. Most silesaurs are characterized by slender limbs and a “beak-like” projection from the anterior tip of the lower jaw^3^. Whereas these reptiles are present in the fossil record of Middle to Upper Triassic^4–8^, no records have been reported from Jurassic or younger strata^9,10^. Silesaurs are particularly interesting because of their close phylogenetic relationships with dinosaurs^4,11,12^, with several studies placing silesaurs as the closest evolutionary relatives of dinosaurs^4,11,13^. On the other hand, there are alternative hypotheses where silesaurs are recovered as members of Ornithischia^9,10,14,15^. This latter scenario supports two main models: (i) silesaurs are part of a monophyletic assemblage (i.e., wide Silesauridae) that is the sister group of “traditional/core ornithischians”^14–16^; or (ii) silesaurs are assembled in low-diversity clades representing a stem group leading to “traditional/core ornithischians”^9,10^. Despite the competing affinities of silesaurs, these reptiles are key taxa in order to understand the dawn of the avian stem lineage. Silesaurs are the oldest dinosauromorphs reported in the fossil record^4,5^, providing clues on the ancestral body plan and biogeography of the group. The Middle Triassic occurrences from Brazil, Tanzania, and Zambia support a gondwanan origin of silesaurs^4,5^, whereas during the Upper Triassic, the group was present in both, Gondwana^17–20^ and Laurasia^7,21,22^. Although restricted to Argentina and Brazil, the fossil record from South America is particularly rich. There are two species from Argentina: *Lewisuchus admixtus*^23^, from the early Carnian beds of Chañares Formation; and *Ignotosaurus fragilis*, from the late Carnian of the Ischigualasto Formation^18^. Regarding Brazil, there are two species too: *Gamatavus antiquus*^8^, from the Ladinian/early Carnian of Santa Maria Formation; and *Sacisaurus agudoensis*^17^, from the early Norian of Caturrita Formation. In addition, there is an unnamed silesaur reported from the mid-to-late Carnian beds of Santa Maria Formation^24^. This unnamed material is remarkable because it comes from the oldest unequivocal dinosaur-bearing beds worldwide^25^, providing evidence of the co-occurrence of distinct groups of Pan-Aves during this crucial moment. Unfortunately, the scarcity of silesaurs from these beds obscures our understanding of the ecological relationships between these groups. Here, we describe the first silesaur species from Carnian (Upper Triassic) beds from Brazil and discuss its role on the evolutionary history of the group.

## Material and methods

### Institutional abbreviations

*CAPPA/UFSM* Centro de Apoio à Pesquisa Paleontológica da Quarta Colônia da Universidade Federal de Santa Maria, São João do Polêsine, Rio Grande do Sul, Brazil; *CRILAR-Pv* Paleontologıa de Vertebrados, Centro Regional de Investigaciones Cientıficas y Transferencia Tecnologica, Anillaco, Argentina; *MNA* Museum of Northern Arizona, Flagstaff, USA; *NMMNH* New Mexico Museum of Natural History and Science, Albuquerque, USA; *UFSM* Laboratório de Estratigrafia e Paleobiologia, Universidade Federal de Santa Maria, Santa Maria, Rio Grande do Sul, Brazil.

### Specimen

The specimens here described are housed at the palaeovertebrates collection of the Centro de Apoio à Pesquisa Paleontológica da Quarta Colônia/Universidade Federal de Santa Maria (CAPPA/UFSM), under the specimen number CAPPA/UFSM 0374 and CAPPA/UFSM 0375.

### Phylogenetic analysis

In order to access the phylogenetic affinities of the new silesaur, it was scored in the data matrix of Norman et al.^10^, which is a modified version of the data matrix published by Müller & Garcia^9^. This is the most comprehensive dataset regarding silesaurs. Furthermore, we inserted *Gamatavus antiquus*^8^, a recently described silesaur from Brazil. Its scoring was performed through first-hand examinations of the holotype (UFSM 11348a, a partial right ilium) and a referred specimen (UFSM 11348b, a partial left femur). *Chilesaurus diegosuarezi* was removed from the data matrix because of its controversial affinities^26^. The most parsimonious trees were recovered in the software TNT v. 1.5^27^. All characters received the same weight and characters 4, 13, 18, 25, 63, 82, 84, 87, 89, 109, 142, 166, 174, 175, 184, 186, 190, 201, 203, 205, 209, 212, 225, 235, 236, 239, 250 and 256 were treated as additive (ordered). *Euparkeria* was used to root the most parsimonious trees, which were constructed using random addition sequence + tree bisection reconnection (TBR), which included 1000 replicates of Wagner trees (with random seed = 0), TBR and branch-swapping (holding 20 trees saved per replicate).

### Femoral length estimation criteria

The total femoral length of CAPPA/UFSM 0374 and CAPPA/UFSM 0375 was estimated according to two ordinary least squares linear regressions employing the dataset of Barrett et al.^28^. This dataset includes measurements of 31 femora of distinct Triassic and Lower Jurassic ornithodirans. The femoral length of CAPPA/UFSM 0374 was estimated using the proximal long axis of the femoral head as the independent variable, whereas the femoral length of CAPPA/UFSM 0375 was estimated using the distal long axis as the independent variable.

## Results

Systematic paleontology

Archosauria Cope, 1869

Pan-Aves Gauthier & de Queiroz, 2001

Dinosauromorpha Benton, 1985

Silesauridae Nesbitt et al., 2010

*Amanasaurus nesbitti* gen. et sp. Nov.

### Holotype

CAPPA/UFSM 0374 (Table 1), a proximal portion of a right femur.

**Table 1:** Measurements (in mm) of the femur of *Amanasaurus nesbitti* gen. et sp. nov.

Specimen	Dimension	Measurement
CAPPA/UFSM 0374	Preserved length	25
CAPPA/UFSM 0374	Estimated total length	121.5
CAPPA/UFSM 0374	Head, maximum anteroposterior width	11
CAPPA/UFSM 0374	Head, maximum transverse width	21
CAPPA/UFSM 0375	Preserved length	36
CAPPA/UFSM 0375	Estimated total length	143.2
CAPPA/UFSM 0375	Distal end, maximum transverse width	26
CAPPA/UFSM 0375	Bone wall	2

### Etymology

The genus combines the Tupi word “amana” (= rain) and the Greek “saurus” (= lizard), referring to the Carnian pluvial episode. The specific epithet honors Dr. Sterling J. Nesbitt, a prominent North American paleontologist, for his contribution and studies on silesaurs and Triassic archosaurs.

### Type locality, age, and horizon

**Figure 1:**
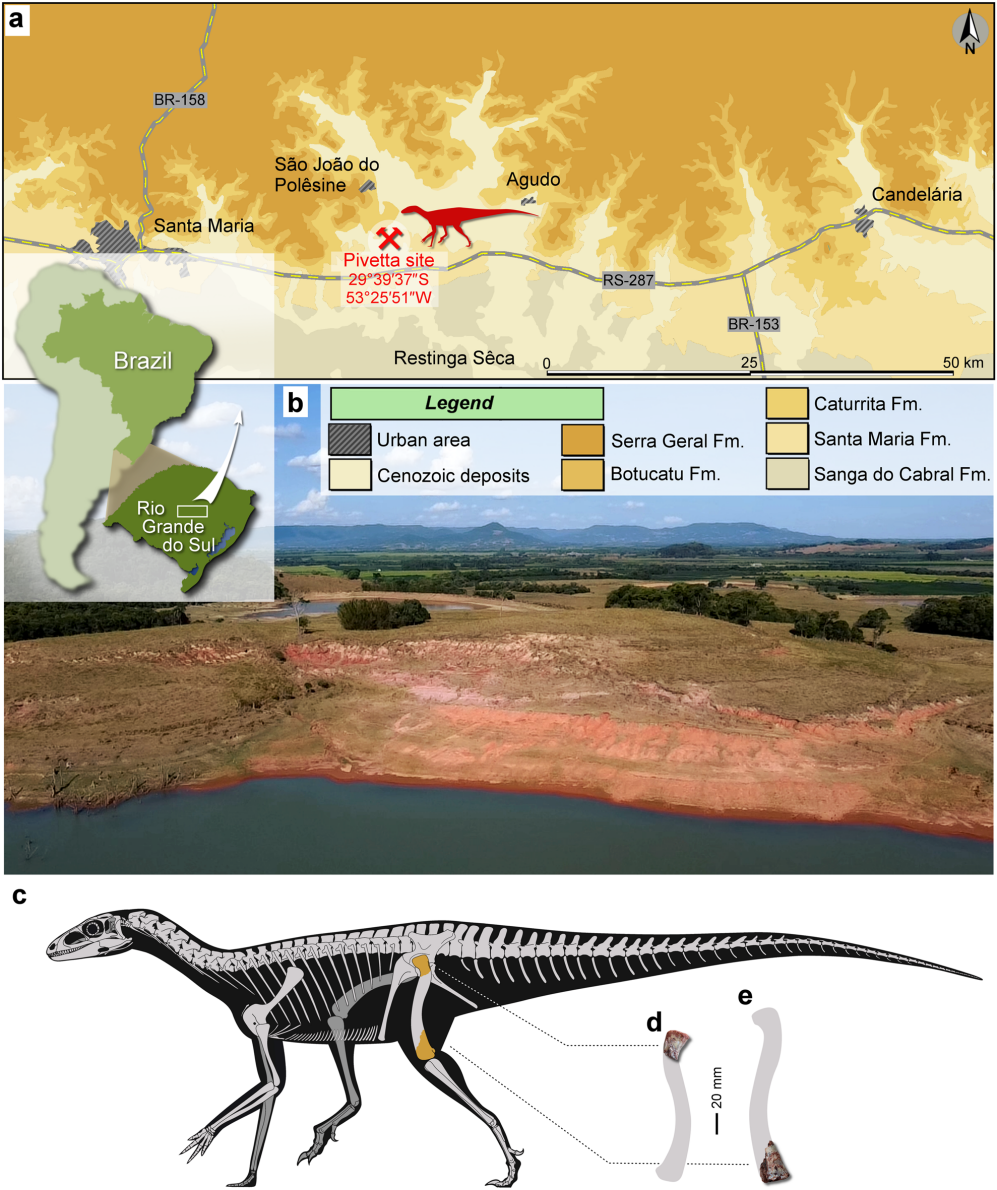
Provenance of *Amanasaurus nesbitti* gen. et sp. nov. (**a**) Surface distribution of the geologic units in the area depicting the location of the Pivetta site. (**b**) General view of the Pivetta site. (**c**) hypothetical reconstruction of the skeleton of *Amanasaurus nesbitti* gen. et sp. nov. depicting (in orange) the preserved portions. (**d**) CAPPA/UFSM 0374 (holotype), a proximal portion of a right femur in anterior view. (**e**) CAPPA/UFSM 0375 (referred specimen), a distal portion of a left femur in anterior view. Figures were generated with GIMP 2.8 (https://www.gimp.org/).

Pivetta site (29°39′37″ S, 53°25′51″ W), between the municipalities of Restinga Sêca and São João do Polêsine, Rio Grande do Sul, Brazil (Fig. 1). Lower portion of the Candelária Sequence^29^ of the Santa Maria Supersequence^30^, Paraná Basin. The presence of the rhynchosaur *Hyperodapedon* places the Pivetta site within the *Hyperodapedon* Assemblage Zone^31^, which is considered mid to late Carnian (Late Triassic) in age according to high-precision U–Pb zircon geochronology that indicated a maximum age of 233.23 ± 0.73 Ma^32^.

### Referred specimen

CAPPA/UFSM 0375 (Table 1), a distal portion of a left femur from an individual slightly larger than the holotype and excavated from the same locality.

### Diagnosis

*Amanasaurus nesbitti* differs from all other known silesaurs with comparable material in (*local autapomorphies): posteromedial tuber of the femoral head reduced to absent; ventral margin of the anteromedial tuber exceeding the femoral head margin; presence of a fossa trochanterica; absence of a raised anterolateral scar; presence of a semi-circular scar on the posterodorsal surface of the femoral head*; cleft between the proximal tip of the anterior trochanter and the femoral shaft (see Supplementary Information for a differential diagnosis).

### Description

**Figure 2:**
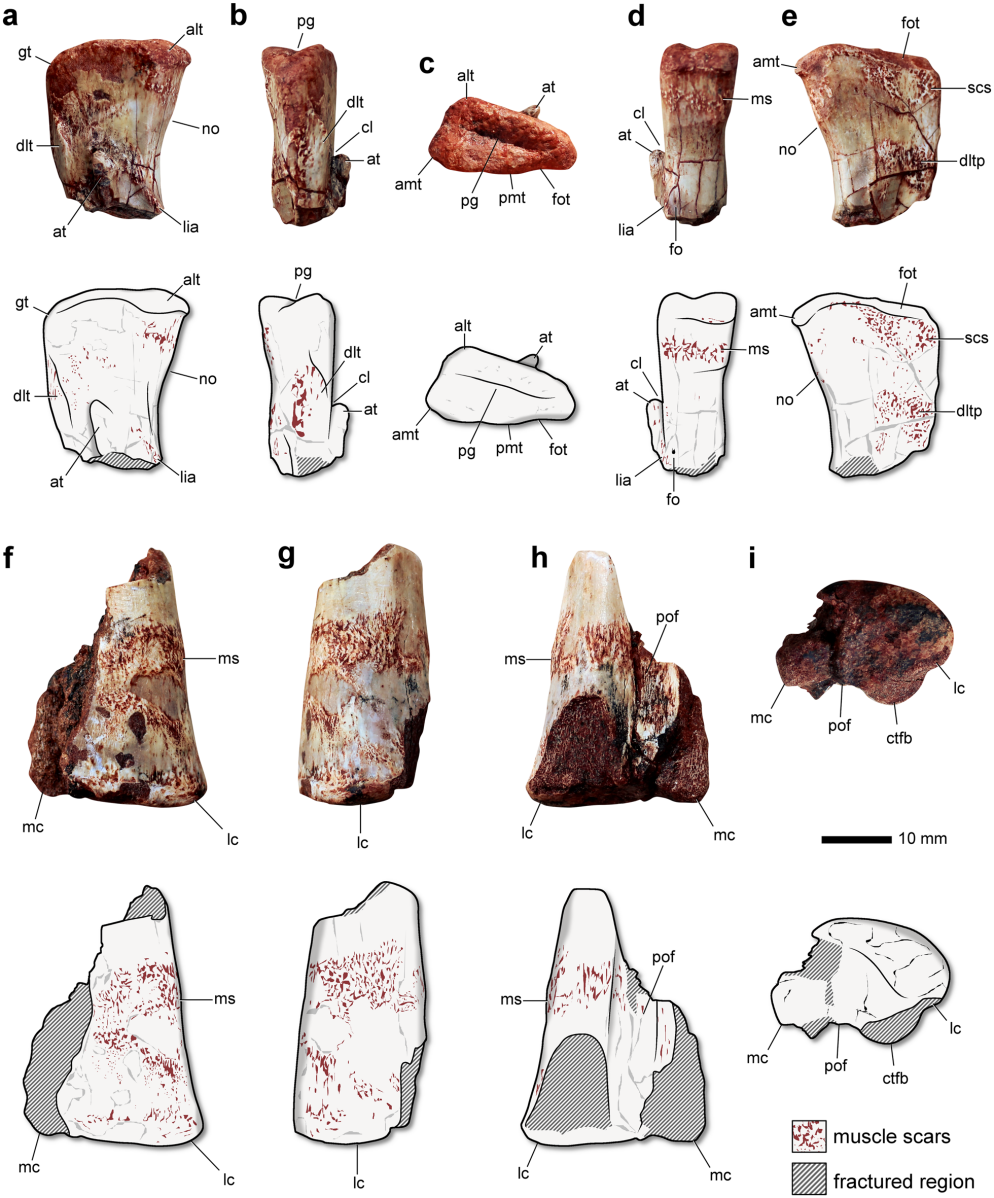
Holotype and referred specimen of *Amanasaurus nesbitti* gen. et sp. nov. from the Candelária Sequence (mid-to-late Carnian) of the Santa Maria Supersequence, southern Brazil. Holotype (CAPPA/UFSM 0374) in anterior (**a**), lateral (**b**), proximal (**c**), medial (**d**), and posterior (**e**) views. Referred specimen (CAPPA/UFSM 0375) in anterior (**f**), lateral (**g**), posterior (**h**), and distal (**i**) views. *alt* anterolateral tuber, *amt* anteromedial tuber, *at* anterior trochanter, *cl* cleft, *ctfb* crista tibiofibularis, *dlt* dorsolateral trochanter, *dltp* posterior portion of the dorsolateral trochanter, *fo* foramen, *fot* fossa trochanterica, *gt* greater trochanter, *lc* lateral condyle, *lia* linea intermuscularis cranialis, *mc* medial condyle, *ms* muscle scar, *no* notch, *pg* proximal groove, *pof* popliteal fossa, *scs* sub-circular scar. Figures were generated with GIMP 2.8 (https://www.gimp.org/).

The femoral head of *Amanasaurus nesbitti* is well-preserved (Fig. 2a–e). The bone surface preserves fine details and the specimen shows no evidence of sedimentary compression. Similar to other silesaurs, the femoral head is poorly expanded transversely. This condition differing the *Am. nesbitti* from most dinosaurs and pterosauromorphs^33,34^. It is triangular in proximal view, with a deep straight groove separating the anterior and posterior surfaces (Fig. 2c). This groove is absent in *Lewisuchus admixtus*^20,34^. The medial articular surface is straight, whereas in *L. admixtus* and *Asilisaurus kongwe* it is rounded^19,35^. The anterolateral tuber is well-developed, as well as the anteromedial tuber. The later forms the posteromedial margin of the femoral head (Fig. 2e), delimiting the distalmost extension of the articular surface of the proximal portion of the femoral head, such as in *Sacisaurus agudoensis*^14^. Distinct from the other tubers, the posteromedial tuber is poorly developed, lacking a sulcus for ligamentum captis femoris in proximal view. The posteromedial tuber of *L. admixtus*, *As. kongwe* and *Eucoelophysis baldwini* is well-developed^19,35^, differing from the new specimen. There is a reduced fossa trochanterica (Fig. 2c), resembling *As. kongwe*^35^. In *L. admixtus* it is well-developed^19^, whereas in other silesaurs it is absent^7,14,21,36^. The greater trochanter is angled, whereas in lagerpetids it is rounded^15,37,38^. The specimen lacks the “overhang structure” on the proximal surface, which is reported for some specimens of *Silesaurus opolensis*^39^. The typical “notch” between the ventral transition from the femoral head to the femoral shaft is present (Fig. 2a). This differs from the concave emargination that marks the transition in most dinosaurs^33^. In addition, the medial articular surface of the femoral head bears a transverse scar above the notch (Fig. 2d). In *Si. opolensis* there is a similar scar that forms the ventral margin of the attachment point of the iliofemoral ligament^40^. There is a smooth surface on the homologous surface of *Sa. agudoensis*^14^. Its surface is reduced in the *Am. nesbitti*. There is an unusual sub-circular scar on the posterior surface of the femoral heart (Fig. 2e), slightly below the fossa trochanterica. An identical scar was not reported for other silesaurs.

The anterior surface of the dorsolateral trochanter is sharp and merges with the shaft well-below the proximal articular surface (Fig. 2a). In contrast, this trochanter is rounded for all the ontogenetic stages sampled for *As. kongwe*^41^. A proximodistally oriented scar runs on the lateral surface of this trochanter (Fig. 2b), whereas a transverse scar extends from the trochanter to the posterior margin of the femoral shaft (Fig. 2e). A raised anterolateral scar is absent, whereas it is reported for *L. admixtus*, *As. kongwe*, and *Si. opolensis*^19,20,39,41^. Indeed, there are faint striations on the homologous surface. The anterior trochanter is finger-like, extending proximodistally. Its proximal tip is separated from the femoral shaft by a cleft (Fig. 2b), such as in several silesaurs, prionodontians, and theropods^10,11,14,42^. On the other hand, in *L. admixtus* and *As. kongwe* the proximal tip merges smoothly with the femoral shaft, lacking the cleft^19,20,35^. Whereas the proximal tip of the anterior trochanter is not connected to the shaft in the new specimen, it is far less expanded than the wing-like trochanter of several prionodontians^10,43,44^. The condition of the new specimen is also distinct from the pyramidal anterior trochanter of some theropods^42,45^. The proximal portion of the linea intermuscularis cranialis rests medial to the anterior trochanter (Fig. 2a). It is absent in *Sa. agudoensis*^14^. A foramen pierces the femoral shaft medial to the proximal portion of the cranial intermuscular line (Fig. 2d). The trochanteric shelf is absent, a condition shared with *Sa. agudoensis*, *E. baldwini*, *Kwanasaurus williamparkeri, Diodorus scytobrachion*, and prionodontians^7,10,11,14,36^.

The referred distal portion of a left femur bears a well-preserved bone surface (Fig. 2f–i). On the other hand, the bone is fragmented. Distinct from lagerpetids^15,37^, the anterior surface is convex in distal view (Fig. 2i), lacking any evidence of an extensor grove. There is a raised scar extending from the anterior to the lateral surface of the bone (Fig. 2f–h), which is common for dinosauromorphs. The lateral margin of the lateral condyle is rounded in distal view and there is a depression between this condyle and the crista tibiofibularis. The exact size and shape of the crista tibiofibularis and the medial condyle are uncertain. Whereas the popliteal fossa is not entirely preserved, it is proximodistally elongated (Fig. 2h), resembling the condition of most silesaurs and aphanosaurs^4,46^. A raised scar runs from the surface above the crista tibiofibularis into the popliteal fossa.

### Phylogenetic analysis

**Figure 3:**
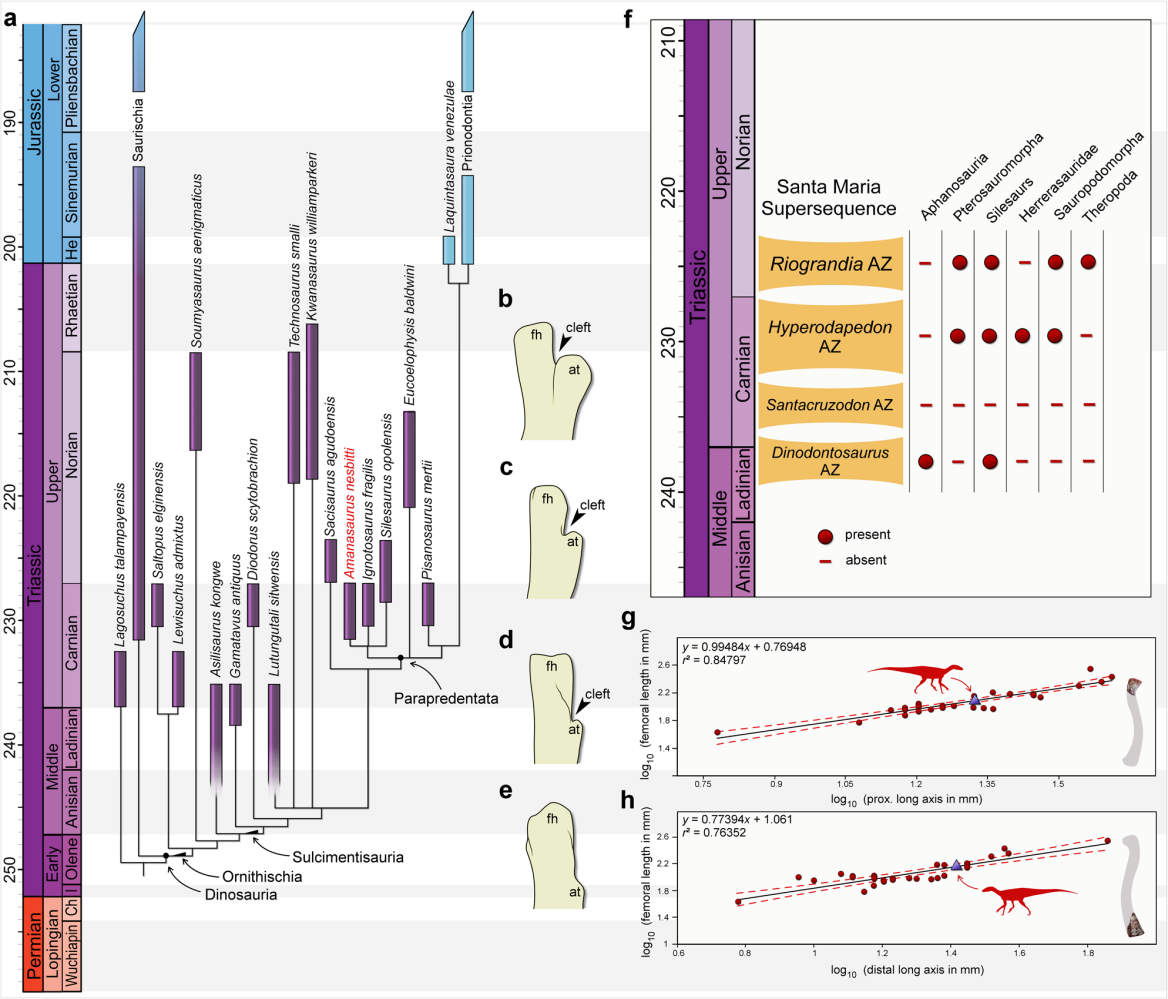
Results of the analyzes. (**a**) Time-calibrated reduced strict consensus tree depicting the phylogenetic position of *Amanasaurus nesbitti* gen. et sp. nov. (**b**) Left (reversed) femur of *Scutellosaurus lawleri* (MNA 175) in lateral view. (**c**) Left (reversed) femur of *Eucoelophysis baldwini* (NMMNH P-22298) in lateral view. (**d**) Right femur of *Amanasaurus nesbitti* gen. et sp. nov. (CAPPA/UFSM 0374) in lateral view. (**e**) Right femur of *Lewisuchus admixtus* (CRILAR-Pv 552) in lateral view. (**f**) Occurrence of Pan-Aves according the Middle to Upper Triassic Assemblage Zones of Brazil; (**g**) Plot of log_10_-transformed proximal long axis of the femur versus log_10_-transformed femoral length of distinct ornithodirans depicting (purple triangle) the estimated femoral length for the holotype of *Amanasaurus nesbitti* gen. et sp. nov. (**h**) Plot of log_10_-transformed distal long axis of the femur versus log_10_-transformed femoral length of distinct ornithodirans depicting (purple triangle) the estimated femoral length for the referred specimen of *Amanasaurus nesbitti* gen. et sp. nov. Figures were generated with GIMP 2.8 (https://www.gimp.org/).

The heuristic search recovered 1728 most parsimonious trees (MPTs) of 1074 steps each, with a consistency index of 0.298 and a retention index of 0.689. The general topology of the strict consensus tree (Fig. 3a) follows that recovered by Norman et al.^10^, where silesaurs are nested in low diversity groups in the branch that leads to Prionodontia (i.e., traditional ornithischians). *Amanasaurus nesbitti* nests as a parapredentatan within Ornithischia in all the MPTs. The new taxon nests in a trichotomy with *Ignotosaurus fragilis* and *Silesarus opolensis*, which is supported by a fossa on the ventral surface of postacetabular part of ilium (ch. 174: 1 → 2), iliac lamina two times deeper or more than the acetabulum (ch. 187: 0 → 1), and ligament sulcus of the femoral head does not form a medial excavation in proximal view (ch. 204: 0 → 1). Only the latter character is coded for *Am. nesbitti*. Following the phylogenetic definition proposed by Nesbitt et al.^4^, the clade supporting *Am. nesbitti*, *I. fragilis* and *Si. opolensis* receives the name Silesauridae.

Regarding *Gamatavus antiquus*, it nests as the basalmost member of Sulcimentisauria, an arrangement supported by the absence of the femoral trochanteric shelf (ch. 215: 0 → 1) and facies articularis antitrochanterica not ventrally descended (ch. 216: 0 → 1). Furthermore, *Saltopus elginensis* nested as sister taxon of *Lewisuchus admixtus* and is recovered as an ornithischian for the first time. This result should be taken carefully, however, given the fragmentary and difficult-to-interpret nature of the holotype. In previous iterations of this dataset *Sa. elginensis* was recovered as an early-diverging saurischian sister to *Eodromaeus murphi*^9^ and as a non-dinosaur dinosauromorph^15^.

## Discussion

The holotype of *Amanasaurus nesbitti* possesses typical traits of silesaurs, such as the presence of a notch between the ventral transition from the femoral head to the femoral shaft and a straight medial articular facet of the proximal portion in proximal view^4,11,14,28,36^. Therefore, *Am. nesbitti* can be safely assigned to a silesaur. Although the referred specimen lacks overlapping material with the holotype, the preserved distal portion of the femur resembles that of other silesaurs (e.g., presence of an elongated popliteal fossa^4^) and lacks typical features of other related groups. So, this specimen is referred to *Am. nesbitti* on the basis of the geological context and the shared morphology with other silesaurs.

Regarding the general morphology of *Am. nesbitti*, it bears a unique set of plesiomorphic and apomorphic traits for silesaurs, which is consistent with its phylogenetic and stratigraphic position. It retains a fossa trochanterica, a trait presents in older silesaurs, such as *Lewisuchus admixtus* and *Asilisaurus kongwe*^19,35^. Conversely, the posteromedial tuber is extremely reduced, resembling the condition observed in late diverging forms, such as in *Sacisaurus agudoensis*^14^ and *Kwanasaurus williamparkeri*^7^. Perhaps, the “transitional” shape of the anterior trochanter comprises one of the most interesting features of the new taxon. The anterior trochanter of dinosaurs and close related groups is usually regarded as the insertion point for the m. iliofemoralis^40,47^. In early silesaurs (e.g., *L. admixtus*; *As. Kongwe; Gamatavus antiquus*), the proximal tip is completely connected to the femoral shaft (Fig. 3e), whereas in late diverging forms (e.g., *Eucoelophysis baldwini*) its tip is completely separated from the shaft by a marked cleft^14^ (Fig. 3c). This condition is interpreted as an early stage of the “wing-like” anterior trochanter of prionodontians (Fig. 3b) and provided further support for the ornithischian affinities of silesaurs^10,14^. In *Am. nesbitti*, the anterior trochanter is less pronounced than in post-Carnian silesaurs; however, it bears the cleft separating the proximal tip from the femoral shaft (Fig. 3d). The new taxon comprises the oldest silesaur expressing this condition, revealing a Carnian origin for this feature.

The new silesaur provides further support for the presence of silesaurs in the *Hyperodapedon* Assemblage Zone (AZ) of Brazil. These reptiles are reported for three of the four AZs assigned to Middle and Upper Triassic^8,17^. The current scenario depicts the total absence of silesaurs and other Pan-Aves solely in the *Santacruzodon* AZ (Fig. 3f), which is poorly sampled in comparison with other AZs and its geographical distribution is limited^29^. In addition, the presence of the new silesaur in Carnian beds of Southern Brazil reinforces the co-occurrence of distinct Pan-Aves groups during the initial evolution of dinosaurs (*ca*. 230 Ma). The new silesaur comes from an outcrop area that yielded lagerpetids, early sauropodomorphs, and herrerasaurids^15,16,48^. This diversity of Pan-Aves surpasses that of older AZs from Brazil (i.e., *Dinodontosaurus* AZ and *Santacruzodon* AZ), being comparable to that of the *Riograndia* AZ (Fig. 3f). A similar diversity is also reported for the coeval Ischigualasto Formation^18^, where a silesaur is also reported (i.e., *Ignotosaurus fragilis*). It is reasonable to conclude that the landscapes that witnessed the early evolution of dinosaurs supported a wide range of avian line archosaurs as well. Moreover, according to the femoral length estimations performed here, *Am. nesbitti* reached the same size of early sauropodomorphs. The estimated femoral length of the holotype (CAPPA/UFSM 0374) is 121 mm (Fig. 3g), whereas for the referred specimen (CAPPA/UFSM 0375) it is 143 mm (Fig. 3h). For comparison, the femoral length of specimens of the early sauropodomorph *Buriolestes schultzi* ranges from 89 mm (ULBRA-PVT056^49^) to 138 mm (ULBRA-PVT280^49^). These specimens were excavated from correlate strata that are 500 m distant from the Pivetta site. It is the first time that silesaurs rivaling in size with early dinosaurs are recovered from the oldest unequivocal dinosaur-bearing beds, challenging the idea that in faunas where silesaurs and unambiguous dinosaurs co-occurred, silesaurs were relatively smaller^28^. This discovery reinforces the complex scenario regarding the radiation of Pan-Aves during the Triassic. Surely, the body plan of early diverging forms being surpassed by late diverging dinosaurs does not fit within the current models anymore. Actually, silesaurs –independent of their phylogenetic position– persisted during most of the Triassic Period, with its plesiomorphic body size advancing through the dawn of dinosaurs, instead of silesaur lineages decrease in body size through time.

### Supplementary Information

Supplementary Information 1.

Supplementary Information 2.

## Data Availability

All data generated or analysed during this study are included in this published article and its Supplementary Information files.
