# Author Correction: A new silesaurid from Carnian beds of Brazil fills a gap in the radiation of avian line archosaurs

**DOI:** 10.1038/s41598-024-70440-4

**Published:** 2024-08-22

**Authors:** Rodrigo T. Müller, Maurício S. Garcia

**Affiliations:** 1 Centro de Apoio à Pesquisa Paleontológica da Quarta Colônia, Universidade Federal de Santa Maria, Rua Maximiliano Vizzotto, São João do Polêsine, Rio Grande do Sul 598, 97230-000 Brazil; 2 Programa de Pós-Graduação em Biodiversidade Animal, Universidade Federal de Santa Maria, Santa Maria, Rio Grande do Sul 97105-120 Brazil

Correction to: *Scientific Reports* 10.1038/s41598-023-32057-x, published online 11 April 2023

The original version of this Article does not include evidence of registration in ZooBank within the work itself, as required by Art. 8.5.3 of the amended Code of the International Commission on Zoological Nomenclature (ICZN)^1^. Therefore, the newly proposed genus-group name *Amanasaurus* and species-group name *A. nesbitti* are not available. This issue has been corrected here and the new taxon name is now available.

The present publication has been registered in ZooBank with the LSID: urn:lsid:zoobank.org:pub:4D9DE818-3DFE-4300-90C4-D65E84DC12B2. The following ‘Zoobank’ and ‘Nomenclatural Acts’ subsections appear below, along with the modified ‘Systematic Paleontology’ section of the original Article^2^.

## Systematic paleontology

Archosauria Cope, 1869

Pan-Aves Gauthier & de Queiroz, 2001

Dinosauromorpha Benton, 1985

Silesauridae Nesbitt et al., 2010

*Amanasaurus nesbitti* gen. et sp. nov.

[urn:lsid:zoobank.org:act:3A0099FE-DC2F-4714-B93E-92A9A1B5C28F (genus)]

[urn:lsid:zoobank.org:act:8250544F-1613–4857-A4ED-684BBB149CFE (species)]

### Holotype

CAPPA/UFSM 0374, a proximal portion of a right femur.

### Etymology

The genus combines the Tupi word “amana” (= rain) and the Greek “saurus” (= lizard), referring to the Carnian pluvial episode. The specific epithet honors Dr. Sterling J. Nesbitt, a prominent North American paleontologist, for his contribution and studies on silesaurs and Triassic archosaurs.

### Type locality, age, and horizon

Pivetta site (29° 39′ 37″ S, 53° 25′ 51″ W), between the municipalities of Restinga Sêca and São João do Polêsine, Rio Grande do Sul, Brazil. Lower portion of the Candelária Sequence^3^ of the Santa Maria Supersequence^4^, Paraná Basin. The presence of the rhynchosaur *Hyperodapedon* places the Pivetta site within the *Hyperodapedon* Assemblage Zone^5^, which is considered mid to late Carnian (Late Triassic) in age according to high-precision U–Pb zircon geochronology that indicated a maximum age of 233.23 ± 0.73 Ma^6^.

### Referred specimen

CAPPA/UFSM 0375, a distal portion of a left femur from an individual slightly larger than the holotype and excavated from the same locality.

### Diagnosis

*Amanasaurus nesbitti* differs from all other known silesaurs with comparable material in (*local autapomorphies): posteromedial tuber of the femoral head reduced to absent; ventral margin of the anteromedial tuber exceeding the femoral head margin; presence of a fossa trochanterica; absence of a raised anterolateral scar; presence of a semi-circular scar on the posterodorsal surface of the femoral head*; cleft between the proximal tip of the anterior trochanter and the femoral shaft (see supplementary information of the original Article for a differential diagnosis).

## Nomenclatural acts

This published work and the nomenclatural acts it contains have been registered in ZooBank, the online registration system for the International Code of Zoological Nomenclature. The ZooBank LSIDs (Life Science Identifiers) can be resolved and the associated information viewed through any standard web browser by appending the LSID to the prefix ‘http://zoobank.org/’. The LSID for this publication is: urn:lsid:zoobank.org:pub:4D9DE818-3DFE-4300-90C4-D65E84DC12B2.
