# Supplementary material for: A new silesaurid from Carnian beds of Brazil fills a gap in the radiation of avian line archosaurs
Source: Sci Rep. 2023 Apr 11;13:4981. doi: 10.1038/s41598-023-32057-x (PMC10090097; doi:10.1038/s41598-023-32057-x)
Supplement: Supplementary file 2 — Supplementary Information 2. [file 41598_2023_32057_MOESM2_ESM.docx]

**SUPPLEMENTARY INFORMATION**

**A new silesaurid from Carnian beds of Brazil fills a gap in the radiation of avian line archosaurs**

Rodrigo T. Müller, Maurício S. Garcia

Corresponding author email: rodrigotmuller@hotmail.com

**Outline of contents:**

1. Differential diagnosis

2. Strict consensus tree

3. Full coding to *Amanasaurus nesbitti* and *Gamatavus antiquus* in the phylogenetic analysis

**1. Differential diagnosis:**

A differential diagnosis based on femoral traits is present below. *Soumyasaurus aenigmaticus*, *Technosaurus smalli*, *Ignotosaurus fragilis* are not listed because these silesaurs lack femoral material.

*Lewisuchus admixtus* differs from the holotype of *Amanasaurus nesbitti* in that it has: medial articular surface of the femoral head rounded in proximal view; well-developed anteromedial and posteromedial tubers; well-developed sulcus for ligamentum captis femoris; lack a proximal groove; lack a transverse scar on the medial surface of the femoral head; raised anterolateral scar; lack a semi-circular scar on the posterodorsal surface of the femoral head; lack a cleft between the anterior tip of the anterior trochanter and the femoral shaft; trochanteric shelf.

*Asilisaurus kongwe* differs from the holotype of *Amanasaurus nesbitti* in that it has: medial articular surface of the femoral head rounded in proximal view; well-developed posteromedial tuber; anteromedial tuber does not exceed the anteromedial margin of the femoral head in anterior/posterior view; lack a transverse scar on the medial surface of the femoral head; raised anterolateral scar; lack a semi-circular scar on the posterodorsal surface of the femoral head; dorsolateral trochanter is not sharp in any sampled ontogenetic stage; lack a cleft between the anterior tip of the anterior trochanter and the femoral shaft; trochanteric shelf.

*Lutungutali sitwensis* differs from the holotype of *Amanasaurus nesbitti* in it has: anteromedial tuber does not exceed the anteromedial margin of the femoral head in anterior/posterior view; well-developed posteromedial tuber; lack a fossa trochanterica; lack a semi-circular scar on the posterodorsal surface of the femoral head; lack a cleft between the anterior tip of the anterior trochanter and the femoral shaft.

*Gamatavus antiquus* differs from the holotype of *Amanasaurus nesbitti* in that it has: medial articular surface of the femoral head rounded in proximal view; well-developed posteromedial tuber; anteromedial tuber does not exceed the anteromedial margin of the femoral head in anterior/posterior view; lack a semi-circular scar on the posterodorsal surface of the femoral head; dorsolateral trochanter is not sharp in any sampled ontogenetic stage; lack a cleft between the anterior tip of the anterior trochanter and the femoral shaft.

*Diodorus scytobrachion* differs from the holotype of *Amanasaurus nesbitti* in that it has: anteromedial tuber does not exceed the anteromedial margin of the femoral head in anterior/posterior view; lack a fossa trochanterica; lack a transverse scar on the medial surface of the femoral head; lack a semi-circular scar on the posterodorsal surface of the femoral head.

*Silesaurus opolensis* differs from the holotype of *Amanasaurus nesbitti* in that it has: “overhang structure” on the proximal surface; anteromedial tuber does not exceed the anteromedial margin of the femoral head in anterior/posterior view; lack a fossa trochanterica; raised anterolateral scar; lack a semi-circular scar on the posterodorsal surface of the femoral head; a trochanteric shelf.

*Sacisaurus agudoensis* differs from *Amanasaurus nesbitti* in that it has: lack a fossa trochanterica; posterior surface of the femoral head with an enlarged smooth surface; lack a semi-circular scar on the posterodorsal surface of the femoral head; and lack a linea intermuscularis cranialis.

*Kwanasaurus williamparkeri* differs from the holotype of *Amanasaurus nesbitti* in that it has: anteromedial tuber does not exceed the anteromedial margin of the femoral head in anterior/posterior view; a ridge running distally from the anteromedial tuber; lack a fossa trochanterica; posterior surface of the femoral head with an enlarged smooth surface; lack a semi-circular scar on the posterodorsal surface of the femoral head; trochanteric shelf.

*Eucoelophysis baldwini* differs from the holotype of *Amanasaurus nesbitti* in that it has: anteromedial tuber does not exceed the anteromedial margin of the femoral head in anterior/posterior view; well-developed posteromedial tuber; lack a fossa trochanterica; lack a semi-circular scar on the posterodorsal surface of the femoral head; enlarged anterior trochanter

**2. Strict consensus tree**

**
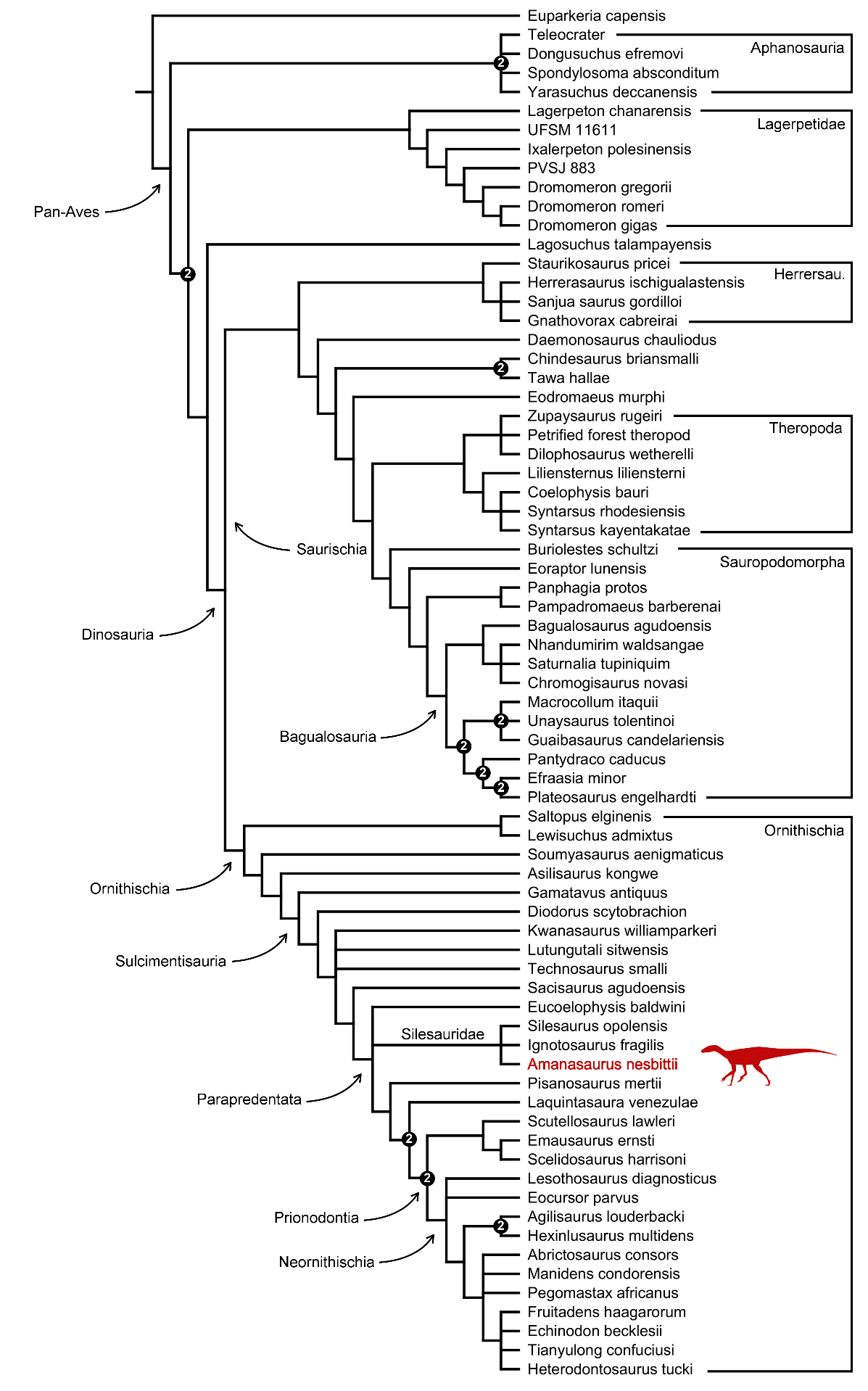
**

**Supplementary Fig. 1.** Strict consensus tree depicting the phylogenetic position of *Amansaurus nesbitti*. Number on nodes represent Bremer support values higher than 1.

**3.** **Full coding to *Amanasaurus nesbitti* and** ***Gamatavus antiquus*** **in the phylogenetic analysis:**

Amanasaurus_nesbitti ??????????????????????????????????????????????????????????????????????????????????????????????????????????????????????????????????????????????????????????????????????????????????????????????????????????101111?0121001111??1?0??10??????????????????????????????????????????????????????

Gamatavus_antiquus ???????????????????????????????????????????????????????????????????????????????????????????????????????????????????????????????????????????????????????????????????????????0020011010?11120???????????????110101?011100011110?????????????????????????????????????????????????????????????
